# Postprandial hyperglycemia and postprandial hypertriglyceridemia in type 2 diabetes

**DOI:** 10.7555/JBR.31.20160164

**Published:** 2017-05-16

**Authors:** Toru Hiyoshi, Mutsunori Fujiwara, Zemin Yao

**Affiliations:** 1. Division of Diabetes and Endocrinology, Department of Internal Medicine, Japanese Red Cross Medical Center, Tokyo, Japan; 2. Department of Laboratory Medicine, Japanese Red Cross Medical Center, Tokyo, Japan; 3. Department of Biochemistry, Microbiology and Immunology, Ottawa Institute of Systems Biology, University of Ottawa, Ottawa, Ontario, K1H 8M5, Canada.

**Keywords:** postprandial hyperglycemia, postprandial hypertriglyceridemia, Type 2 diabetes mellitus, atherosclerosis

## Abstract

Postprandial glucose level is an independent risk factor for cardiovascular disease that exerts effects greater than glucose levels at fasting state, whereas increase in serum triglyceride level, under both fasting and postprandial conditions, contributes to the development of arteriosclerosis. Insulin resistance is a prevailing cause of abnormalities in postabsorptive excursion of blood glucose and postprandial lipid profile. Excess fat deposition renders a vicious cycle of hyperglycemia and hypertriglyceridemia in the postprandial state, and both of which are contributors to atherosclerotic change of vessels especially in patients with type 2 diabetes mellitus. Several therapeutic approaches for ameliorating each of these abnormalities have been attempted, including various antidiabetic agents or new compounds targeting lipid metabolism.

## Introduction

Type 2 diabetes mellitus (T2D) is an important global health problem. Microvascular complication, such as retinopathy or nephropathy, is common in poor glycemic control patients. Furthermore, increased risk of cardiovascular disease is obvious in T2D patients, which is attributable to endothelial dysfunction. In patients with hyperglycemia, oral glucose loading suppressed endothelial-dependent vasodilation through an increase in the production of oxygen-derived free radicals. Epidemiological studies, such as DECODE (Diabetic Epidemiology: Collaborative Analysis of Diagnostic Criteria in Europe), have shown that accelerated postprandial blood glucose elevation is strongly associated with occurrence of cardiovascular diseases^[1]^. Postprandial glucose level is an independent risk factor for cardiovascular disease that has effects greater than glucose level of fasting state^[2]^. The DECODE study showed a direct relationship between 2-h glucose levels (in oral glucose tolerance test) and risk for cardiovascular death^[3]^.

Generally, management of LDL cholesterol (LDLc) in serum lipids is an important strategy in arteriosclerotic disease prevention, which has been suggested from the results of a large number of epidemiological studies and large-scale clinical trial. Cohort study in Japan showed a significant correlation between triglyceride (TG) values and coronary artery disease^[4–5]^. On the other hand, it has been reported^[6]^ that increase of serum triglyceride level, both fasting and postprandial state, is involved in arteriosclerosis. The widely accepted concept of postprandial hyperlipidemia was initially proposed by Zilversmit *et al*. in 1979^[7]^.

The pathophysiology of postprandial hyperglycemia is characterized by hyperglycemic spikes that induce oxidative stress. Postprandial hyperglycaemia is defined as a plasma glucose level>7.8 mmol/L (140 mg/dL) 1-2 hours after ingestion of food^[8–9]^. In contrast, postprandial glucose level in people with normal glucose tolerance is less than 7.8 mmol/L (140 mg/dL) in response to meals and typically returns to premeal levels within two to three hours. However, because of ethnic gap in physique (expressed in body mass index or BMI), the East Asian people in general are relatively small in physique compared to African and Caucasian. Meta-analyses of insulin sensitivity index (SI) and acute insulin response to glucose (AIRg) in three major ethnic groups (i.e. 19 African, 31 Caucasian, and 24 East Asian cohorts) have shown divergent natural courses of diabetes onset among different ethnic groups^[10]^. Nevertheless, the incidence of progression of atherosclerotic disease appears to coincide with any of the pathological conditions of hyperglycemia and dyslipidemia. In this article, we discuss various aspects of postprandial hyperglycemia and postprandial hyperlipidemia in T2D^[11–16]^.

## Postprandial hyperglycemia (see ***Fig. 1***)

Postprandial excursion of blood glucose level is dependent upon several rate-limiting factors, including (i) time course of gastric emptying, (ii) intestinal absorptive rate of glucose, (iii) decreased insulin sensitivity in peripheral tissues, (iv) decreased suppression of hepatic glucose output (glycogenolysis) after meals, (v) rate of gluconeogenesis of the liver, (vi) insulin secretion rate during postprandial period, and (vii) autonomic nerve imbalance of sympathetic and parasympathetic nerve.

**Fig.1:**
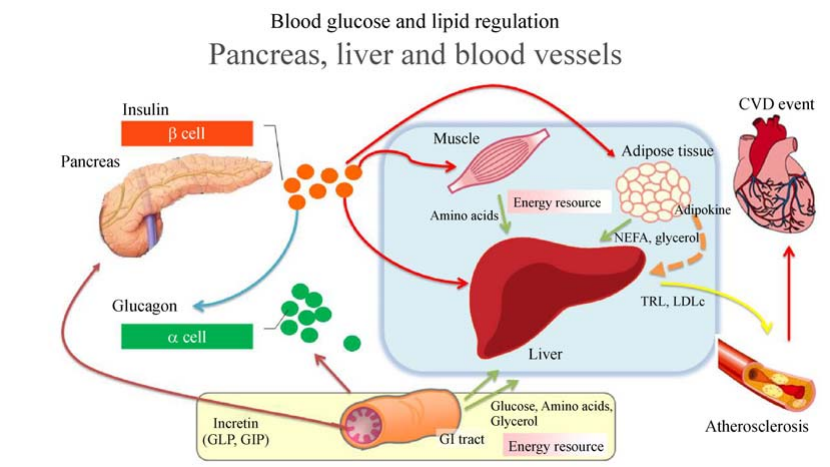
**Blood glucose and lipid regulation. pancreas, liver and blood vessels. ** After meal, pancreatic beta cells release insulin to inhibit hepatic gluconeogenesis and glycogenolysis. Insulin also acts at peripheral tissues to increase glucose uptake (muscle), contributing to decreased blood glucose level. GLP-1 and GIP are incretin hormones which secreted by the gut. Incretin hormones can stimulate pancreatic beta cells to secrete insulin. GLP-1 suppressed inappropriate glucagon secretion from pancreatic alpha cells. Nutrient from the gastrointestinal tract, muscle and adipose tissue such as glucose, amino acids and glycerol which are energy resource of the body introduce into the liver. In insulin resistance state, overproduction of TRL associated with hepatic VLDL production. Increasing TRL is strongly contribute hypertryglyceridemia which is related indirectly to atherosclerotic progression through increase in sdLDL and lowered HDL. Interaction between enlarged fat cell and macrophages in adipose tissue evokes chronic inflammatory response, resulting in overproduction of FFA.

Postprandial excursion of plasma glucose level is a common phenomenon in people with diabetes. For example, in a study in which daily plasma glucose profiles was assessed over a one-week period in 3,284 subjects with non-insulin-treated T2D, postprandial plasma glucose value>8.9 mmol/L (160 mg/dL) was recorded at least once in 84% of those studied^[17]^. Deterioration of β-cell function and insulin secretion are prior to clinical diabetes. These metabolic abnormalities are first evident by elevations in postprandial plasma glucose^[18]^.

Blood glucose levels mainly regulate the balance of input and output the liver (i.e. gluconeogenesis and glycogenolysis). The liver is chiefly responsible for this glucose homeostasis. The kidneys are also contributing an average 20% of glucose release, and the gut supplying up to 15 to 20%. During fasting state, pancreatic α-cells secrete glucagon to increase hepatic gluconeogenesis and glycogenolysis, resulting in an increase in circulating glucose levels. After meal, pancreatic β-cells release insulin to inhibit hepatic gluconeogenesis and glycogenolysis, thus decreasing glucose output to the blood stream. Insulin also acts at peripheral tissues to increase glucose uptake, contributing to decreased blood glucose levels. In patients with T2D, insulin action is decreased at the liver and/or peripheral tissue, whereas the glucagon action is increased. As a result, T2D is invariably associated with increased hepatic gluconeogenesis and glycogenolysis, increased glucose output to the circulation, repressed glucose uptake into the peripheral tissues, and eventually increased blood glucose levels.

Prior to clinical diabetes, the above metabolic abnormalities are first evident by elevated concentration of postprandial plasma glucose. Emerging evidence shows that postprandial plasma glucose levels are elevated due to deficiencies in amylin, a glucoregulatory peptide that is normally co-secreted with insulin from the β-cells, as well as glucagon-like peptide-1 (GLP-1) and glucose-dependent gastric inhibitory peptide (GIP), which are incretin hormones secreted by the gut^[19–22]^. Released during absorption of meals, the intestine-derived incretin hormones GLP-1 and GIP can stimulate pancreatic β-cells to secrete insulin. It is estimated that GLP-1 and GIP are responsible for 50%–70% of postprandial insulin release^[23]^. In addition, GLP-1 suppresses inappropriate glucagon secretion from pancreatic α-cells, and at pharmacologic doses, GLP-1 delays gastric emptying by inhibiting gastroduodenal motility^[24]^, which is associated with an increase in satiety and reduced food intake. Both GLP-1 and GIP are rapidly broken down by DPP-4 after secretion^[25]^.

Postprandial hyperglycemic state includes production of AGEs and lipid peroxidation products as activators of upstream kinase such as protein kinase C (PKC) and p38α mitogen activated protein kinase (MAPK), resulting in endothelial dysfunction and inflammatory genes response^[26–27]^. Oxidative stress plays an important role for vascular endothelial dysfunction. Endothelial dysfunction of vessels is characterized by decreased endothelium-derived relaxing factor such as nitric oxide (NO) and endothelial NO synthase (eNOS)^[28]^. Oxidative stress also contributes to progression of atherogenesis, including proliferation of smooth muscle cells and formation of vascular plaque^[29–30]^.

The superoxide anion is produced in mitochondria through univalent reduction of molecular oxygen. Xanthine oxidase, NADH/NADPH oxidase, lipoxygenase and NOS are key enzymes in the process of generation of superoxide anions. Superoxide anions are reduced to hydrogen peroxide by enzymatically catalyzed dismutation. Among reactive oxygen species (ROS), hydroxyl radical is generated by hydrogen peroxide and a transition metal catalyzed reaction (Haber-Weiss reaction). Hydroxyl radical has extremely high reactivity and is involved in a number of tissue damage, including DNA damage, lipid peroxidation, and protein degeneration that are associated with progression of atherosclerosis^[31]^. Nitrotyrosine and 8-iso-prostaglandin F2α (8-iso-PGF2α), two strong oxidative stress markers, are increased in postprandial hyperglycemia. A strong positive correlation between urinary 8-iso-PGF2α and glycemic variability assessed by MAGE (mean amplitude of glycemic excursions) has been described^[32–33]^.

It is well known that inflammation is closely related to the pathogenesis of atherosclerosis and vascular failure^[12,34–36]^. Inflammatory cells and mediators play an essential role in the initiation and progression of atherosclerosis. Various circulating adhesion molecules, such as intracellular adhesion molecule 1 (ICAM-1) and vascular adhesion molecule 1 (VCAM-1), are increased in diabetes with or without vascular disease^[34]^. These molecules are strongly related to the recruitment of monocytes and T-lymphocytes to the endothelium of the artery wall, resulting in activation of early inflammatory process. Other adhesion molecule, for instance β2-integrin Mac1 (CD11b/CD18), binds to endothelial surface ICAM-1 and platelets through interacting with either fibrinogen or several platelets receptors, such as glycoprotein Ib-α (GP Ib-α) and ICAM2^[36]^.

Firth *et al*.^[37]^ have measured, using multiple tracer approach, the rate of appearances of glucose, either derived from the ingested glucose or from the endogenous glucose production. It was found that people with T2D showed no increase in the appearance of the ingested glucose as compared with normal subjects. However, the rate of appearance of endogenous glucose production was increased in T2D patients as compared to normal subjects. These data suggest that delayed postprandial excursion of glucose in T2D patients is not attributable to over-absorption of ingested glucose. Rather, increase in the rate of glucose appearance is mainly attributable to hepatic insulin resistance.

T2D has been considered as a bi-hormonal disease characterized by relative hypoinsulinemia and hyperglucagonemia^[38]^. Patients with T2D display postprandial hyperglycemia due to decreased insulin secretion and a concomitant increase in glucagon secretion. The uncontrolled glucagon release under postprandial stage under T2D conditions may not be attributable to lack of an insulin action, because experiments with an α-cell-specific insulin receptor knockout mouse (αIRKO) model showed that insulin exerts no direct effect on glucagon secretion from α-cells^[39]^.

Longitudinal monitoring of insulin and glucagon secretion in Caucasian women with impaired glucose tolerance (IGT) versus normal glucose tolerance (NGT), over a 12-year period, showed that β- and α-cell dysfunction are evident several years before diagnosis of IGT, and islet dysfunction is manifested by impaired glucose sensitivity of the β- and α-cells and reduced maximal insulin secretion^[40]^.

Besides impaired β-cell functions, a lowered number of β-cell mass may also contribute to insufficient secretion of insulin in these patients. Analysis of autopsy samples of pancreas from 50 T2D subjects showed that β-cell mass in pancreas was 36% lower than that of 52 non-diabetic subjects^[41]^. While the topography of α and β-cells was similar in both groups, the ratio of α/ β cell areas increased (from 0.42 to 0.72) in T2D subjects whereas the α-cell mass was virtually identical^[41]^. Thus, the high proportion of α- to β-cells in the islets of some T2D subjects is due to decrease in β-cell number rather than increase in α-cell number. This imbalance may lead to the relative hyperglucagonemia observed in T2D.

Recently, it has been suggested that hepatic insulin resistance is associated with several molecules that are related to insulin signal modulation. Of which, regulation of IRS-1 and IRS-2 were observed in diabetic models^[42,43]^. IRS-1 acts mainly in fasting stage whereas IRS-2 acts mainly under postprandial conditions. Further, it was suggested that a sufficiently high level of IRS-2 expression in the liver during fasting state (i.e. between meals) is important in suppressing postprandial hyperglycemia. Thus, suboptimal level of IRS-2 expression, often observed under hyperinsulin-secretion conditions (e.g. obesity or taking snacks besides meals), was thought to be a contributing factor to postprandial hyperglycemia.

### -Vascular complications in diabetes due to persistent hyperglycemia and postprandial hyperglycemia. (see ***Fig. 2***)

Diabetic vascular disorders encompass microvascular and macrovascular disorders. The microangiopathy is mainly diabetic retinopathy and diabetic nephropathy, while the macrovascular disorder includes myocardial infarction, cerebral infarction and lower extremity arteriosclerosis. The effects of chronically sustained hyperglycemia are related to the progression of microvascular complications. In the Diabetic Control and Complications Trial (DCCT), it was proved that strict blood glucose level control is important for the onset and progression of retinopathy and diabetic nephropathy^[44]^. The DCCT study was a comparative analysis between subjects with type 1 diabetes mellitus who received intensive insulin treatment and those receiving conventional treatment. Evidence for the onset and suppression of progression of microvascular complications by glycemic control is also shown in UKPDS 33^[45]^ and Kumamoto study^[46]^. It is thought that factors, such as consistent hyperglycemia by polyol pathway, activation of protein kinase C, increased accumulation of glycated protein, and enhancement of oxidative stress, contribute to in vascular endothelial dysfunction.

**Fig.2:**
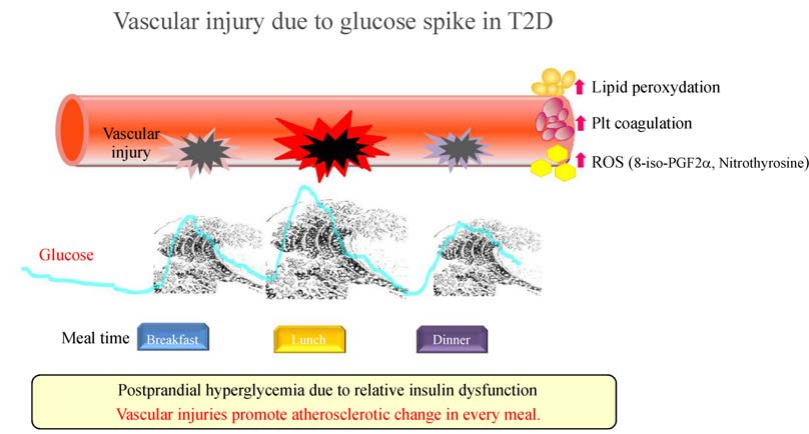
**Vascular injury due to glucose spike in T2D.** The rapid fluctuation in blood glucose level repeated every meal is called "glucose spike", which damages blood vessels and causes progression of atherosclerosis. In this condition, increasing lipid peroxydation, platelet coagulation and synthesis of ROS. Arteriosclerosis starts from the early stage of postprandial hyperglycemia of diabetes, or the stage of IGT earlier than that.

On the other hand, macroangiopathy is not specific to diabetes mellitus, and diabetes mellitus is one of the risk factors for vascular disorders. For example, it has been reported that contributing risk factors to the onset of coronary artery disease in type 2 diabetes are LDLc, HDLc, HbA1c, systolic blood pressure, and current smoking status^[47]^. A study of Japanese type 2 diabetic patients also confirmed that the risk factors for coronary artery disease are triglyceride, LDL cholesterol, HbA1c, and systolic blood pressure^[4]^. In comparison with diabetic microvascular complications, it is suggested that the involvement of dyslipidemia is greater than the blood glucose risk expressed by HbA1c in macrovascular complications.

From the viewpoint of blood glucose control in diabetes, postprandial blood glucose level is more relevant than fasting blood glucose level to the development and progression of macroangiopathy. The DECODE study has shown that accelerated postprandial excursion is strongly associated with occurrence of cardiovascular diseases^[3]^. In Funagata Diabetes Study, it was demonstrated that even a mild rise in postprandial blood glucose is associated with cardiovascular death^[48]^. Similarly, the results of the DECODA Study targeting Asian races reported that blood glucose levels are important for prediction of total death and cardiovascular death after 2 h of glucose tolerance^[49]^. In the result of meta-analysis that integrated results of seven studies that tracked over 52 weeks in acarbose and placebo group for T2D, it was reported that the relative risk of onset of myocardial infarction was significantly reduced to 64% and the relative risk of onset of systemic vascular events was reduced by 35%^[50]^. On the other hand, basic research also showed that glucose spike (rapid increase in blood glucose) provokes endothelial cell apoptosis and induces endothelial cell damage^[51]^. Therefore, as a goal of blood glucose control of diabetic patients, it is necessary to control postprandial hyperglycemia to suppress the development of diabetic macrovascular diseases.

Therefore, the phenomenon of postprandial blood glucose excursion in T2D is similar to the effect of sustained increase in blood glucose with fasting blood glucose level in microvascular complications. While in diabetic macrovascular complications, postprandial blood glucose excursion is considered to be more specifically involved than sustained hyperglycemia.

### -Postprandial hypertriglyceridemia

Postprandial hypertriglyceridemia refers to a state in which serum TG shows abnormally high value after meals and its peak is delayed / prolonged. Diagnosis of dyslipidemia is generally carried out in “early morning fasting” for more than 12 hours. T2D is one of the most common diseases associated with elevation in serum triglyceride levels^[52–56]^. Hypertriglyceridemia contributes to progression of arteriosclerosis indirectly through increased small dense low density lipoproteins (sdLDL) and deceased high density lipoproteins (HDL). Hypertriglyceridemia is attributable to abnormalities in the synthesis and catabolism of triglyceride-rich lipoproteins (TRL), such as very low density lipoprotein (VLDL) and chylomicrons. Structural protein of TRL is apoB100 or apoB48. ApoB100 is produced in the liver parenchymal cells and apoB48 is produced in intestinal enterocytes. Overproduction of TRL is mainly associated with hepatic VLDL production. Insulin resistance is an important factor that regulates hepatic TRL production. Under normal condition, hepatic apoB100 synthesis is regulated by intracellular degradation that limits the level of VLDL assembly and secretion^[11]^. Intracellular degradation of apoB100 is controlled by insulin action. In insulin resistance state, overproduction of apoB100-VLDL occurs and, as a consequence, hypertriglyceridemia ensures. There are two types of VLDL produced by human liver, namely VLDL_1_ and VLDL_2_^[55]^. Hepatic overproduction of VLDL in insulin resistant state, such as diabetes and metabolic syndrome, is strongly related to VLDL_1_, whereas the level of VLDL_2_ production remains relatively normal under this condition. The observed VLDL_1_ overproduction could at least be partially attributable to increased blood glucose levels^[57]^.

## Postprandial hypertriglyceridemia as a risk factor of atherosclerosis

Postprandial hypertriglyceridemia is a state in which the peak of TG increase after meals is high as well as a state where TG does not return to pre-meal levels. The chylomicron remnant concentration is increased in postprandial hyperlipidemia as compared with that in the fasting state. The association between increased and prolonged postprandial hypertriglyceridemia and coronary artery disease has been reported^[58–59]^.

Analysis of Multiple Risk Factor Intervention Trial showed that greater prevalence of hypertriglyceridemia with nonfasting than fasting values, and similarly increased risk with each indicates that nonfasting TG levels may be more useful than fasting ones for risk stratification^[60]^. Iso *et al.* also reported that in the group with high TG under non-fasting condition, there is high risk of cardiovascular disease, coronary artery disease such as myocardial infarction and onset of sudden death^[6]^.

## Apo C-III function in triglyceride metabolism

Recently, the apoC-III functionality has resurged as a main topic in lipid and lipoprotein metabolism^[61]^. ApoC-III is a small protein (79 amino acids) abundantly presented in the plasma as a component of TRLs and HDL. The plasma triglyceride and apoC-III concentrations are positively correlated with each other in normo- and hypertriglyceridemic subjects. ApoC-III has been shown to slow down the clearance of TRLs by inhibiting the activity of LPL and by interfering with binding to cell-surface receptors. Human studies with familial hyperchylomicronemia patients (averaging TG at 1406 to 2083 mg/dl) have shown that introducing mRNA of apoC-III resulted in reduction in serum triglyceride levels^[62–64]^. Patients with loss-of-function mutations in the *APOC3* gene exhibited low risk of cardiovascular events compare with wild type control subjects^[65]^. The risk of CHD with carriers of any *APOC3* mutations was 40% lower than the risk of the non-carriers. The low risk of cardiovascular events in patients with loss-of-function mutations in the *APOC3* gene is strongly related to serum low triglyceride levels.

A recent study has reported a positive correlation between apoC-III protein levels and plasma fasting glucose level and glucose excursion in overweight patients^[66]^. It was also shown that high blood glucose concentration plays a role in rat and human apoC-III expression through the action of transcription factors ChREBP and HNF-4α^[66]^. These data suggest that apoC-III may be one of the regulatory factors contributing to hypertriglyceridemia (overproduction of TRL) under elevated glucose concentration conditions.

Biosynthesis of TRL requires MTP (microsomal transfer protein), which is an endoplasmic reticulum resident heterodimeric complex. The expression of apoB gene and its serum level under diabetic condition are related to upregulated MTP, which has been demonstrated in various animal models^[67–69]^. In human T2D, increased expression of MTP mRNA in intestinal biopsies was shown^[70–71]^. T2D patients who were in statins had lower MTP mRNA compared to the controls. Hepatic MTP mRNA expression is negatively regulated by insulin. Insulin might also directly inhibit apoB48 secretion even though it is probable that upregulation of MTP stimulates apoB secretion^[72–73]^.

## Association of hyperglycemia and hypertriglyceridemia for diabetic vascular complication

Elevation of serum triglyceride is not caused by insulin deficiency, but is often associated with a relative decrease in insulin action (i.e. insulin resistance). Insulin resistance mainly shows that the insulin action in liver and skeletal muscle is lower than that in healthy subjects. In insulin resistance state, overproduction of apoB100-VLDL occurs and hypertriglyceridemia ensures.

Diabetic patients have a high probability of becoming dyslipidemia, and when diabetes and dyslipidemia are merged, they increase the risk of cardiovascular disease. A meta-analysis showed that the relative risk of developing coronary artery disease due to diabetes is 2.0 and the risk of developing cerebral infarction is 2.3^[74]^. According to NIPPONDATA 80, the relative risk of death in coronary artery disease patients with diabetes is 2.8^[75]^. In Hisayama-chyo study, the relative risk of developing coronary artery disease after diversification of confounding factor in diabetic patients is 2.6 and the relative risk of developing cerebral infarction is 3.2, which is higher than those of normal glucose tolerance group^[76]^.

Hyperglycemia can be a factor that triggers hypertriglyceridemia in diabetic patients. When the blood glucose level rises in diabetic patients, the liver uses excess glucose to make triglyceride. Since lipoprotein lipase (LPL) is activated by insulin, triglycerides tend to accumulate when secretion of insulin deteriorates due to diabetes. On the other hand, hypertriglyceridemia exacerbates insulin action (insulin resistance), and blood glucose level further exacerbates diabetes. Diabetes promotes arteriosclerosis, but arteriosclerosis progresses faster when dyslipidemia is combined.

Many epidemiological, experimental and clinical studies have been performed to determine the incidence of cardiovascular disease and metabolic disorders related to hyperglycemia and hyperlipidemia^[6,48,77–83]^. Dyslipidemia is strongly related to and an important risk factor of cardiovascular disease in T2D. Result of the STENO2 study (a long-term follow-up for incidence of vascular complications in diabetic patients)^[84]^ have indicated that not only blood sugar control but also optimal blood pressure and lipid control is needed to achieve the reduction of cardiovascular events. Hypertriglyceridemia contributes indirectly to arteriosclerosis progression through increase in sdLDL and lowered HDL.

Cardiovascular risk factors are present in overlap with those in patients with T2D, such as obesity, hypertension, and hypertriglyceridemia. Moreover, the presence of postprandial abnormalities, namely postprandial hyperglycemia and postprandial hypertriglyceridemia, are the most important inter-related risk factors for the development of cardiovascular disease in patients with T2D.

Insulin resistance is closely related to both postprandial excursion of blood glucose and lipid profile. Clinical manifestation of insulin resistant status is obesity or metabolic syndrome. Inflammatory process is present in these conditions. IL1β and IL6 are the major inflammatory cytokines that stimulate expression of sterol regulatory element binding protein 2 (SREBP2) and 3-Hydroxy-3-Methylglutaryl Coenzyme A (HMG-CoA) in HepG2 cells^[85–87]^. Upregulation of SREBP2 through extracellular signal regulated pathways involves the kinases ERK-1 and -2.

There is a strong relationship between metabolism of free fatty acid (FFA) in adipocytes and that in liver cells. Histological view of adipose tissue in patients with obesity shows crown-like structures (CLS) that are the appearances of fat cells and surrounding macrophages. Interaction between enlarged fat cell and macrophages evoked chronic inflammatory response, resulting in overproduction of FFA. Increase influx of FFA into the liver can lead to fatty liver and non-alcoholic steatohepatitis (NASH)^[88–89]^.

Macrophages in the liver can be activated by degenerated hepatocyte. Phagocytosis and digestion of degenerated hepatocyte by macrophages may result in chronic inflammatory change and liver fibrosis. Deposition of FFA in the liver is also the cause of insulin resistance and postprandial hyperglycemia^[90]^.

## Therapeutic approach of postprandial hyperglycemia and hyperlipidemia

Currently, various antidiabetic agents are being developed for the treatment. Since onset of cardiovascular disease can be prevented by suppressing postprandial excursions of blood glucose level, various drugs have been used clinically. There is some evidence showing that suppression of cardiovascular events can be achieved by using a single antidiabetic agent. For instance, the STOP-NIDDM study^[91]^ has shown that cardiovascular events can be reduced by the treatment of acarbose, an α-glucosidase inhibitor.

### Drugs for specific suppression of postprandial hyperglycemia (see ***Table 1***)

Intestinal absorption of the glucose depends on total gut function. α-Glucosidase (maltase, α-glucopyranosidase, α-glucoside hydrolase, α-1,4-glucosidase) is a glycoside hydrolase located in the brush border of the small intestine. α-Glucosidase breaks down complex carbohydrates such as starch and glycogen into their monomers, thus plays a role in glucose absorption. The cleavage occurs at α-1,4-glycosidic bond between individual glucosyl residues from various glycoconjugates, including α- or β-linked polymers of glucose.

**Tab.1:** Current drugs for glucose regulation

Drug	Target	Clinical effects/ availability	Effect for PPHG/ PPHL	Limitation	Refs
Metformin	Unclear; involves complex I & mGPD	Extensively used	Fasting HG	GI side effect Nausea, Diarrhea	
α-glucosidase inhibitor	Intestinal α-glucosidase	Reduction of CVD	PPHG	GI side effect Constipation, Farting	49,91,93
Glinides	β cells Pancreas	Short duration of action	PPHG	Use every meal time	98
DPP4inhibitor	Intestinal DPP4	Suppression of glucagon	Fasting/PPHG	GI side effect Nausea, Constipation	100-102
Thiazolidinedione	Liver, Fat PPAR γ	Increase in insulin sensitivity	Fasting HG	Edema, Bone fracture	
Sulfonylureas	β cells pancreas	Insulin stimulator	Fasting HG	Prolonged hypoglycemia	96,97
SGLT2 inhibitor	Renal tubular SGLT2	Decrease CVD outcome Mild body weight reduction	Fasting HG	Urogenital infection Dehydration	
Insulin (Insulin analogue)	Insulin receptor	Robust glucose reduction Extensively used	Fasting/PPHG	Increase body weight Injection	44
GLP1 agonist	α/β cells pancreas	Reduce satiety Mild body weight reduction	PPHG	Nausea, Vomiting Injection	99

mGPD: mitochondrial glycerol-3-phosphate dehydrogenase; HG: hyperglycemia; GI: gastro intestinal; CVD: cardiovascular disease; PPHG: post prandial hyperglycemia; DPP4: dipeptidyl peptidase 4; PPAR γ: peroxisome oroliferator-activated receptor γ.

In humans, the pancreas and salivary gland synthesize amylase (α-amylase) that hydrolyses dietary starch into disaccharides and trisaccharides. The secretion from the Lieberkuhn glands of the small intestine contains the digestive enzymes maltase, lactase, and sucurase. These digestive enzymes are abundantly distributed in the vicinity of microvilli of intestinal epithelial cells. Thus, nutrients that have been digested near microvilli can be absorbed efficiently by the cells. Starch blockers are substances that inhibit amylase. It has been demonstrated that concentrated starch blocker extracts from white bean (Phaseolus vulgaris), when given with a starchy meal, can reduce the usual rise in blood glucose levels of both healthy subjects and diabetics^[92]^.

Clinically, only α-glucosidase inhibitors (e.g. acarbose, voglibose and miglitol) are used to inhibit postprandial blood glucose excursion. Daily dose of voglibose at 0.2mg orally can reduce 2 h postprandial blood glucose excursion by 2.0+/- 2.15 mmol/L in T2D patients^[93]^. Adverse events associated with the use of these compounds include diarrhea, constipation and farting.

Postprandial hyperglycemia is partially dependent of the rate of insulin secretion during postprandial period, which is believed to be associated with genic predisposition of T2D patients. Insulin secretion from the pancreatic β- cells is composed of two phases, the first fast phase is followed by a second relatively slow phase in response to rapid rise in blood glucose concentrations^[94]^. The early phase of insulin secretion is important for the rapid and efficient suppression of endogenous glucose production after a meal.^[95]^ In patients with T2D, the first phase of insulin secretion is reduced, and the reactivity of the second phase is delayed.

Sulfonylureas stimulate insulin secretion from pancreatic beta cells mediated by the sulfonylurea receptor that displays a high affinity toward sulfonylureas. Glibenclamide is the most powerful among the sulfonylurea agents. At daily dose of 1.25-2.5 mg of oral administration, the duration of glibenclamide action is 12-18 hours and halflife (T_1/2_) is 2.7 hours. On the other hand, Glimepiride is a relatively weak insulin secretagouge, thus exerting a mild hypoglycemic effect, yet its insulin sensitivity enhancing effect is almost the same as glibenclamide. Duration of glimepiride action is 6-24 h (T_1/2_ = 1.5 hours). However, these compounds are not specifically effective for reducing postprandial glucose excursion^[96–97]^.

Glinides, a group of drug that lead to insulin secretion in an immediate and short-term, bind to the sulfonylurea receptor transiently. Although the glucose lowering effect of glinides is weak compared to the other sulfonylurea agents, the effect appears in about 15 min after taking and reaches maximum blood concentration in about 30 min. Therefore, glinides can reduce postprandial hyperglycemia and improve the first phase insulin secretion^[98]^.

Incretin hormones, such as GLP-1 and GIP, not only are insulin secretagouge but also exert other actions in regulation of blood glucose level, such as decreasing motility of GI tract that can delay gastric emptying. GLP-1 can also suppress inappropriate glucagon secretion by pancreatic α-cells and therefore decrease endogenous hepatic glucose production by approximately 50%. The glucagon secretion may otherwise contribute to reduction of postprandial glucose excursion.

A pharmacological approach to control blood glucose level in T2D patients is the use of dipeptidyl peptidase 4 (DPP-4) inhibitor and GLP-1 receptor agonist. These agents are strongly effective for reduction of postprandial glucose excursion. GLP-1 receptor agonists (e.g. exenatide, liragrutide, lixisenatide) are synthetic DPP-4 resistant form of endogenous GLP-1. For example, exenatide has 53% homology with endogenous GLP-1 and longer circulating halflife than that of endogenous GLP-1^[99]^. Exenatide binds to the GLP-1 receptors on pancreatic β-cells and augments glucose-induced insulin secretion.

DPP-4 inhibitors decrease the metabolism of GLP-1 and GIP through inhibition of the enzyme DPP-4. In normal physiologic conditions, the DPP4 enzyme rapidly inactivates GLP-1 and GIP by cleaving the two end-terminal amino acids of these incretin hormones. DPP-4 inhibitors increase prandial insulin secretion and suppress glucagon secretion. Postprandial glucose level of patients with T2D decreases by decreasing hepatic glucose production and improving peripheral glucose uptake. DPP-4 inhibitors (e.g. alogliptin, anagliptin, linagliptin, saxagliptin, sitagliptin, teneligliptin and vildagliptin) are currently approved for treatment of T2D^[100–102]^.

### Drug for suppressing hyperlipidemia in diabetes (see ***Table 2***)

Long-term blood glucose control can prevent the onset of microvascular complications such as diabetic nephropathy and diabetic retinopathy, as well as inhibit the worsening of microvascular complications. Strict blood glucose control per se, however, cannot reduce the incidence of the onset of cardiovascular disease^[103]^. However, a large number of evidence showed that lipid-improving drugs, especially cholesterol-lowering, can achieve cardiovascular disease prevention^[5,104–105]^.

**Tab.2:** Drugs for hyperlipidemia in diabetes

**Drug**	**Target**	**Clinical effects** **/availability**	**Effect for** **TC/TG**	**Limitation**	**Refs**
Statin	HMG-CoA reductase	Extensively used Reduction of CVD	Both	Increase CK Rhabdomyolysis	5,104, 105
Fibrate	PPARα	Reduction of CVD	TG	Rhabdomyolysis	107
Ezetimibe	intestinal NPC1L1 PPAR δ/β	Short duration of action	TC	GI side effect	
Ecolocumab	PCSK9	Familial hypercholesterolemia or high risk CVD patients	TC	Need regular injection	108,109
ISIS 304801	Antisense inhibition ApoC-III	TG reduction 31.3-70.9% Under clinical trial	TG	Need regular injection	62,63
CP-346086 JTT-130	MTP inhibitor	Underdevelopment	TG		110,111
Ginko biloba	Lipoprotein(a) synthesis inhibition	Supplementary use	TC		114-117
Tocilizumab	IL6 inhibition	Patients for rheumatoid arthritis	TC		118-121
Torcetrapib Anacetrapib, Evacetrapib	CETP inhibitor	Underdevelopment (anacetrapib, evacetrapib)	TC	Blood pressure and serum aldosterone increased	122

List of the drugs for hyperlipidemia in diabetes mellitus. Only Statin, Fibrate, Ezetimibe and Evolocumabhave already widely in clinical use. TC: total cholesterol; TG: triglyceride; CVD: cardiovascular disease; CK: creatinine kinase; GI: gastro intestinal tract.

Although cardiovascular prevention studies suggests that both statins and fibrate can improve lipid profiles, a clear clinical evidence that treatments focusing on postprandial triglyceride rise can prevent cardiovascular disease remains to be established^[106]^. Currently, there is no new agent specifically effective for postprandial excursion of triglycerides. The combination therapy of fibrate and statin for total serum lipid profile improvement has been studied. The effectiveness of statins in the prevention of cardiovascular diseases has been widely recognized. Studies examining the lipid improvement effect of fibrates have demonstrated that reduction in frequency of cardiovascular diseaseas compare with control group^[107]^.

The results of epidemiological studies conducted in Japan indicate that the triglyceride levels, along with the blood glucose level, are significant cardiovascular risk factors in Japanese diabetic patients^[4]^. However, despite management of triglyceride level is important in patients with T2D, evidence on the effectiveness of management standards and drug therapy is scarce.

Type IIb dyslipidemia, a combination of hypercholesterolemia and hypertriglyceridemia, has been treated with statin in conjunction with lifestyle modification. Combination therapy of fibrates and statins has not been widely used in general clinical situation because of the risk of rhabdomyolysis or elevation of serum creatin kinase.

Recently, new approaches that target the inhibition of proprotein convertase subtilisin/kexin type 9 (PCSK9) have been developed to increase the removal of atherogenic lipoproteins from plasma^[108]^. The PCSK9 inhibitor (e.g. evolocumab) can markedly reduce serum LDL cholesterol concentrations. No severe adverse event has occurred in the clinical trial of evolocumab therapy. But there is the necessity of subcutaneous injection of evolocumab every 1-2 weeks^[109]^.

New type of intervention using antisense inhibition of apoC-III is effective for reduction of serum triglyceride level from 31.3% to 70.9% in a dose-dependent manner^[63–64]^.

Other agents are under development, such as inhibition of the synthesis of apo B, inhibition of MTP^[110–111]^, inhibition of adenosine triphosphate citr-ate lyase to inhibit the synthesis of cholesterol^[112–113]^, inhibition of the synthesis of lipoprotein(a) by inhibition of Interleukin-6 (IL-6) signaling with natural compounds (e.g. Ginko biloba)^[114–117]^ or the IL-6 receptor antibody Tocilizumab^[118–121]^.

Inhibition of cholesteryl ester transfer protein (CETP) has the potential of reduction of serum lipid level^[122]^. These agents are developed for general dyslipidemia patients and not for specific dyslipidemia patients with diabetes, nor are they developed for postprandial hyperlipidemia patients.

### Future pharmacological modulation for improving postprandial hyperglycemia and hyperlipidemia

Agents that target at the stage of gluconeogenesis and glycolysis in the liver have been developed as new antidiabetic drugs. Many of them (e.g. sulfonylurea, biguanide) show effects on reducing the fasting blood glucose level. However, whether or not they specifically suppress postprandial hyperglycemia is not clear. Some of the agents (e.g. thiazolidinedione) are also associated with triglyceride metabolism, thus improving effect on postprandial hyperlipidemia is expected. The antidiabetic agents act upon one of the three aspects in the liver: (i) glucose metabolism, (ii) pyruvate flux^[123]^, and (iii) gluconeogenesis enzymes. The compounds under development, yet not in clinical use, for modulating liver glucose metabolism include activator of glucokinase^[124–125]^, inhibitors of FBPase^[126]^, inhibitor of PTP-1B^[127]^, inhibitors of glycogen phosphorylase^[128–129]^, and glucagon receptor antagonist^[130]^. Glucagon receptor antagonists lead to a blood sugar lowering effect by suppressing excessive glucagon secretion in T2D. There is a possibility that glucagon receptor antagonists can suppress postprandial hyperglycemia.

Insulin has been found to decrease hepatic glucose production by suppressing pyruvate flux through inhibition of adipose lipolysis^[131]^.

Pyruvate carboxylase inhibition is one the targets of reduce hyperglycemia. Experimental model of the diabetic rats resulted in lowered blood glucose level and rates of gluconeogenesis. It also revealed decreased adiposity and hepatic steatosis^[123]^.

Another approach to inhibit pyruvate flux is blocking pyruvate transport across the inner mitochondrial membrane into the mitochondrial matrix, which is facilitated by the transport complex composed of mitochondrial pyruvate carrier 1 (MPC1) and MPC2. The MPC inhibitor UK-5099 can suppress glucose production in primary hepatocytes and increase glucose uptake in myocytes.^[132–133]^

Targeting gluconeogenesis enzyme, such as phosphoenolpyruvate carboxylase (PEPCK) and glucose-6-phosphatase (G6Pase), is another approach to reduce blood glucose level. Pyruvate is converted to oxaloacetate by pyruvate carboxylase in mitochondria. PEPCK catalyzes conversion of oxaloacetate to phosphoenolpyruvate (PEP) in cytosol. Gluconeogenesis substrates include lactate (which is changed to pyruvate by lactate dehydrogenase) and amino acids. Glycerol can also enter gluconeogenesis pathway through conversion to fructose-1,6-bisphosphate. PEP is converted into fructose-1,6-bisphosphate, which is then converted into fructose-6-phosphate (catalyzed by fructose-1,6-biphosphatase (FBPase)) and subsequently into glucose-6-phosphate (catalyzed by phosphohexose isomerase). G6Pase catalyzes the conversion of glucose-6-phosphate to glucose. PEPCK expression is dysregulated and increased in diabetes. PEPCK is the rate-limiting enzyme for gluconeogenesis and has been implicated as a potential target to reduce blood glucose level. 3-mercaptopicolonic acid inhibits PEPCK and results in hypoglycemia^[134]^. But patients with T2D do not have elevation in liver PEPCK and G6Pase. Therefore, achieving a complete inhibition of PEPCK or G6Pase per se may not be sufficient to reduce blood glucose levels.

Targeting transcriptional factors and co-activators (e.g. PCG1-α, FOXO, and CREB) could potentially be an effective method for treatments T2D. For example, FOXO proteins have been shown to regulate hepatic lipid metabolism. However, transcription factors are frequently found in multiprotein complexes, and designing small molecules that potently change the activity of these multi-protein complexes can be difficult. Nevertheless, direct FOXO1 inhibition was successful in decreasing fasting blood glucose and triglyceride levels in db/db mice^[135]^. Thus, modification of transcriptional factors and co-activators is very attractive as pharmacological interventions may achieve both blood glucose level control and lipid profile improvement in T2D patients. However, for clinical use, it is necessary to establish hepatic specificity of these interventions, because these transcriptional factors and co-activators are also involved in many other processes in different cells and tissues.

Another strategy for controlling glucose metabolism in the liver is enhancing glucose utilization through mitochondrial uncoupling that dissipates the proton gradient across the mitochondrial inner membrane. The uncoupling compounds, such as 2,4-dinitrophenol (DNP), have been developed and tested in animal models. Administration of DNP to diabetic rats decreased fasting plasma levels of glucose, triglycerides, and insulin^[136]^. Controlled-release mitochondrial protonophore (CRMP) is an orally available version of DNP, which has been shown to effectively lower plasma glucose, triglycerides and insulin in wild type or Zucker diabetic fatty rats fed a high-fat diet^[137]^.

The aforementioned compounds all have the potential to reduce blood glucose and/or triglyceride levels in an experimental setting. However, it remains to be determined whether these substances are effective for suppressing postprandial level of blood glucose or triglycerides.

For patients with insulin resistant status such as obesity and metabolic syndrome, partial agonist for adiponectin receptor is probably effective to suppress postprandial hyperglycemia. AdipoRon (adiponectin receptor agonist) is a small molecular compound that binds to adipoR1 and adipoR2 receptor at a low micromolar concentration^[138–140]^. Like adiponectin, adipoRon activates 5′-adenosine monophosphate–activated protein kinase (AMPK) in cultured mammalian cells, an enzyme that is involved in many metabolic processes including release of insulin, inhibition of lipid synthesis, and stimulation of glucose uptake. AdipoRon also activates the transcriptional coactivator peroxisome proliferator–activated receptor gamma coactivator 1–α (PGC1α), which boosts mitochondrial proliferation and energy metabolism. These effects are probably useful for improvement in both glucose metabolism and lipid metabolism.

## Conclusion

Postprandial hyperglycemia and postprandial hypertriglyceridemia are both contributors to atherosclerotic change of vessels especially in T2D patients, the process mediated by oxidative stress and inflammatory change of adipose tissue, liver and vascular wall. One of the key substrates is free fatty acid in adipose tissue. Deposition of excess fat in adipose tissue depends on over-nutrition and sedentary life style in contemporary civilized situation, which creates a vicious cycle of hyperglycemia and hypertriglyceridemia in postprandial state. Specialized therapeutic approaches of postprandial hyperglycemia and hyperlipidemia have not been sufficient to reduce risk of cardiovascular event and other complication of T2D patients. New types of pharmacological agents are essential to the resolution of this clinical problem.
